# Adipokines Expression and Effects in Oocyte Maturation, Fertilization and Early Embryo Development: Lessons from Mammals and Birds

**DOI:** 10.3390/ijms21103581

**Published:** 2020-05-19

**Authors:** Anthony Estienne, Adeline Brossaud, Maxime Reverchon, Christelle Ramé, Pascal Froment, Joëlle Dupont

**Affiliations:** 1 INRAE UMR 85 Physiologie de la Reproduction et des Comportements, F-37380 Nouzilly, France; anthony.estienne@inrae.fr (A.E.); adeline.brossaud@inrae.fr (A.B.); christelle.rame@inrae.fr (C.R.); pascal.fromen@inrae.fr (P.F.); 2 CNRS UMR 7247 Physiologie de la Reproduction et des Comportements, F-37380 Nouzilly, France; 3 Université François Rabelais de Tours, F-37041 Tours, France; 4 Institut Français du Cheval et de l’Equitation, Centre INRAE Val de Loire, F-37380 Nouzilly, France; 5 SYSAAF-Syndicat des Sélectionneurs Avicoles et Aquacoles Français, Centre INRAE Val de Loire, F-37380 Nouzilly, France; maxime.reverchon@inrae.fr

**Keywords:** oocyte maturation, early embryo development, adipokines, mammals, birds

## Abstract

Some evidence shows that body mass index in humans and extreme weights in animal models, including avian species, are associated with low in vitro fertilization, bad oocyte quality, and embryo development failures. Adipokines are hormones mainly produced and released by white adipose tissue. They play a key role in the regulation of energy metabolism. However, they are also involved in many other physiological processes including reproductive functions. Indeed, leptin and adiponectin, the most studied adipokines, but also novel adipokines including visfatin and chemerin, are expressed within the reproductive tract and modulate female fertility. Much of the literature has focused on the physiological and pathological roles of these adipokines in ovary, placenta, and uterine functions. The purpose of this review is to summarize the current knowledge regarding the involvement of leptin, adiponectin, visfatin, and chemerin in the oocyte maturation, fertilization, and embryo development in both mammals and birds.

## 1. Introduction

In mammals and birds, the female reproductive functions are dependent on nutritional status and body composition. Indeed, extreme body weight changes affect human fecundity through increasing the probability of anovulation. For example, a rapid decline in fecundity was shown during the Second World War when a military blockade resulted in a fall in rations to the population [1]. In birds, an excessive food consumption in comparison to their needs in immature females leads to an accelerated development of the reproductive system at the time of sexual maturity [2]. This excess of energy decreases the production of functional oocytes due to a dysregulation of the follicular hierarchy [3]. Thus, as in mammals, an alteration of both nutritional status and body composition is associated with reproductive disorders and embryo development failures in birds. This connection between metabolic and reproductive functions involves many signals called energy sensors that bind specific receptors on the cell surface. Among them are adipokines, which are cytokines that are predominantly produced by white adipose tissue (WAT) and include leptin, adiponectin, visfatin/PBEF (Pre-B-cell colony-enhancing factor), and chemerin. In addition to the WAT, some of these adipokines are present and released by endothelial, immune, and reproductive cells. They play important roles in metabolic homeostasis, cellular inflammation, immunity, angiogenesis, and reproductive functions [4,5]. Indeed, some in vitro and in vivo evidence shows that adipokines are able to control ovarian steroidogenesis, late embryo development, including embryo implantation, and fetal growth [4,6,7,8,9,10]. In mammals, many of these adipokine effects have been described in several reviews [4,6,7,8,9,10,11,12]. However, the roles of leptin, adiponectin, visfatin, and chemerin in oocyte maturation, fertilization, and early embryo development (cleavage and blastocyst formation (before implantation in mammals)) have rarely been summarized.

In the present review, we will focus on these four adipokines that have been the most studied in chicken.

## 2. Oocyte Maturation, Fertilization, and Early Embryo Development in Mammals

### 2.1. Oocyte Maturation and Fertilization

**Figure 1:**
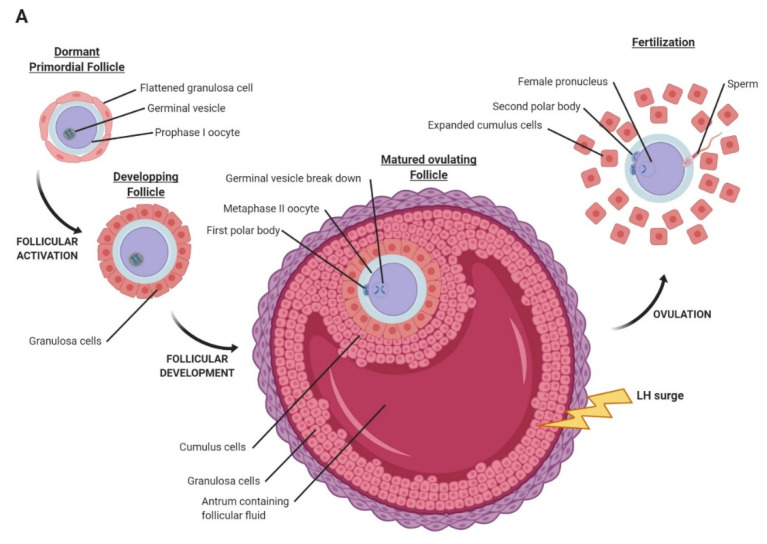

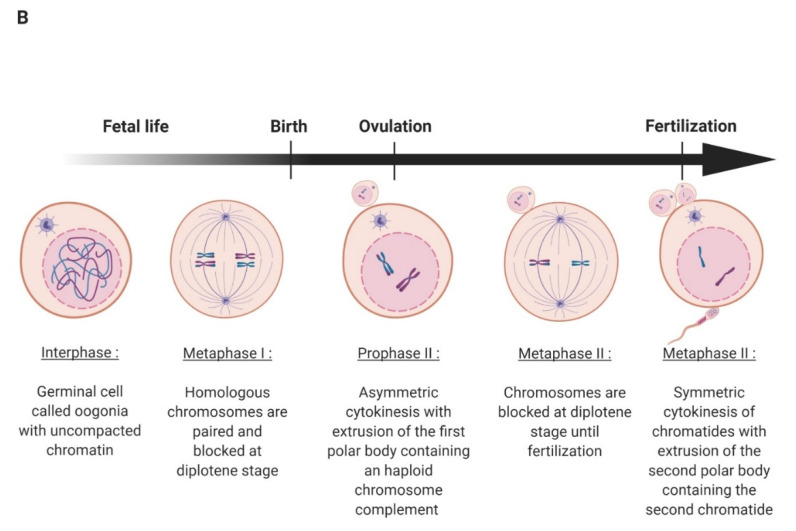
(**A**) Oocyte maturation and fertilization in mammals. (**B**) Description of the different steps of oocyte development from fetal life, birth, ovulation to fertilization.

In many mammalian species, the first meiosis of the oocyte is initiated during fetal life and is arrested at the diplotene stage of prophase before birth (Figure 1A,B). Oocyte maturation is defined as the reinitiation and completion of the first meiotic division, subsequent progression to metaphase II, and the nuclear and cytoplasmic processes which become essential for fertilization and early embryo development (Figure 1A,B). During these processes, the oocyte has undergone extensive growth in cellular interaction with the granulosa and theca cells. The oocyte undergoes asymmetric cytokinesis and extrudes the first polar body containing a haploid chromosome complement (Figure 1A,B). The first meiotic division is completed, and the second meiotic division is initiated, but oocytes arrest in metaphase II until contact with a spermatozoon (Figure 1A,B). The initiation of maturation in fully grown oocytes present in antral follicles is based on the mid-cyclic onset of the luteinizing hormone (LH) surge or the external administration of human chorionic gonadotropin (hCG). Mechanisms of oocyte maturation in vivo and in vitro are still under investigation.

The journey of Early Embryo Development (EED) begins at ovulation when a LH surge induces oocyte expulsion from the follicle and oocyte maturation accompanied by first polar body formation. This step belongs to EED, since the oocyte is not in direct physical contact with the female organism. At this point, the oocyte is mature and able to be fertilized by a single spermatozoon. For this crucial phenomenon, the zona pellucida of the oocyte plays a very important role as a mediator of the oocyte–spermatozoon interaction. Indeed, this glycoprotein layer surrounding the oocyte is formed during folliculogenesis. It is composed of four main types of glycoproteins called Zona Pellucida glycoproteins (ZP) or ZP1, ZP2, ZP3 and ZP4 in humans and mice. These glycoproteins have a critical role in fertilization since they take part in the interaction between the two gametes. In mice, ZP glycoprotein-3 (ZP3) acts as the primary sperm receptor and ZP glycoprotein-2 (ZP2) as the secondary sperm receptor. In humans, ZP glycoprotein-1 (ZP1), ZP3, and ZP glycoprotein-4 (ZP4) bind to the capacitated human sperm and induce acrosomal reaction. ZP2 binds to the acrosome-reacted human spermatozoa [13]. The acrosomal reaction is the release of the contents of the acrosome that reorganize the zona pellucida and permit the spermatozoon to reach the oocyte. This reaction also induces modifications of the ZP proteins that are very important to avoid polyspermia [13,14]. At these stages, the embryo is still surrounded by the zona pellucida to avoid ectopic implantation. The fertilized oocyte, now called a one cell zygote, undergoes second polar body extrusion to remove its remaining set of extra chromosomes. Subsequently, the maternal and the paternal pro-nuclei, each containing haploid genomes, appear and migrate towards one another and fuse during a process called syngamy [15].

### 2.2. Cleavage and Blastocyst Formation 

**Figure 2:**
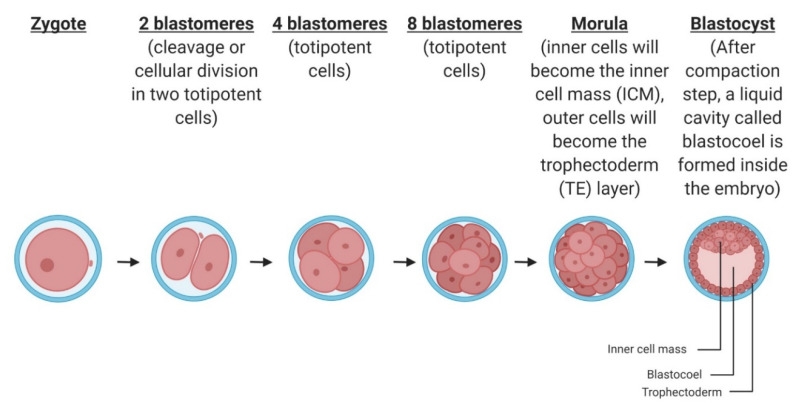
Different steps of the early embryo development in mammals.

Following syngamy, the zygote undergoes a series of mitotic cell divisions (cleavages) that occur within the oviduct and that produce an exponential number of progressively smaller cells called blastomeres (Figure 2), without changing the total volume of the embryo [16]. Data related to cleavage rates in vitro for the human [17,18] and mouse [19] are available and show an average cell cycle time of 13–16 and 10 h, respectively, over the first three or four cleavage divisions. In general, mouse zygotes reach the fully expanded blastocyst stage after 84–96 h of culture and human embryos take another 24–30 h. The average diameter of mouse embryos at 70 μm is about half that of human embryos, thus making the volume of the human embryo ~8-fold larger than the mouse embryo. These mitotic divisions continue until the morula stage, when the embryo cells start the process of intracellular adhesion called compaction. This step is very important since it will affect future cell specification or cellular morphogenetic events [20,21]. The timing of compaction is different between rodents and large mammals such as ovines, porcines, bovines, and primates including humans. Indeed, compaction occurs earlier in rodents at the 8 cells stage instead of 16 to 32 cells for the large animals [17,22,23,24]. Until the morula stage, blastomeres are homogenous and considered as totipotent cells potentially giving embryonic cells or extraembryonic cells. The compaction process will induce the apparition of two cellular subpopulations during the following division. The first population will divide symmetrically according to the inner/outer axis to give two polarized daughter cells. The second population will divide asymmetrically on a perpendicular axis to the previous one to produce an external polarized cell and an internal non-polarized cell. Therefore, at this stage of development, two cell types, different from their inner or outer positions, compose the embryo. While the cells on the inside of the embryo will become a part of the inner cell mass (ICM), the cells on the outside will contribute to the trophectoderm (TE) layer. From the compaction step at day 3 in mice and later at day 4 for other mammals, a liquid cavity called blastocoel is formed inside the embryo. Because of this delay, the human embryos are also likely to undergo at least one additional round of cell division to form a 256-cells blastocyst (Figure 2), whereas mouse blastocysts typically comprise 164 cells. At this stage, the embryo is composed of the external layer of TE, the blastocoel and the ICM. The mouse embryo reaches this developmental step at day 4. Similar to the human species, the majority of the mammals like rat, rabbit, ovine, porcine, and primate species reach the multicellular blastocyst stage on day 5 to day 7 [25,26,27,28]. For bovine species, the embryo does not reach the blastocyst stage until day 7 to day 9 [29]. In preparation for implantation into the uterus, the blastocyst then ‘hatches’ from the ZP to allow for increased embryo growth and development as well as TE adhesion to the lining of the uterine wall. This latter step occurs at day 4.5 in mice, day 7 for the majority of mammals and around day 20 in bovine species [30].

## 3. Oocyte Maturation, Fertilization, and Early Embryo Development in Birds

### 3.1. Oocyte Maturation

In most avian species, the female retains only the left part of her reproductive system. Indeed, in birds except raptors, the right ovary of females, present at hatching, gradually regresses. Thus, only the left ovary is functional in adulthood. Despite a different follicle location between birds and mammals, the maturation process of the ovarian follicles is similar in chickens and mammals. 

**Figure 3:**
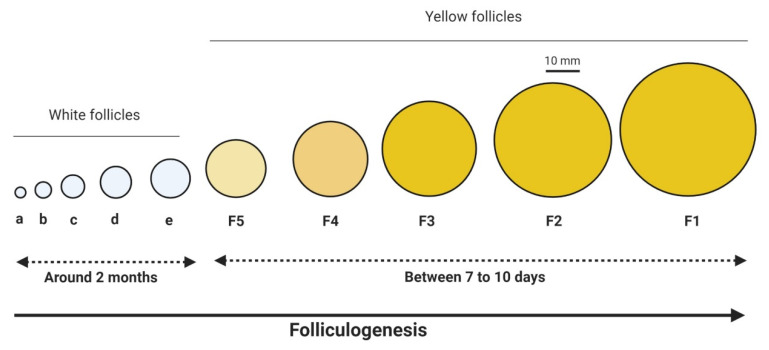
Different steps of the folliculogenesis in birds.

The mature avian ovary contains follicles of different sizes and developmental stages, including primordial follicles, prehierarchical growing follicles (1–4 mm) called white follicles, and large yolk-filled follicles (9–40 mm) recruited to the preovulatory hierarchy [31] (Figure 3). In the primordial follicle, the oocyte contains a large and prominent germinal vesicle (oocyte nucleus). In the yolk-filled follicle, the oocyte contains a large amount of yolk and is surrounded by the perivitelline layer (equivalent to the mammalian ZP) [32]. This perivitelline layer starts to appear between the plasma membrane of the oocyte and the granulosa cell layer during the growth of follicles. The germinal disc containing the oocyte nucleus and cytoplasmic organelles is located at the animal pole. One of the peculiarities of the avian follicle is the presence of 5–7 hierarchical follicles before ovulation. Like the mammalian oocyte, the avian oocyte is arrested in prophase of meiosis I during follicular development. It resumes from meiosis I a few hours before ovulation. However, it is again arrested in metaphase of meiosis II until ovulation. The mature oocyte from the largest yolk-filled follicle (F1, 40 mm) is released into the infundibulum and becomes the ovulated ovum or the egg.

The follicle is surrounded by perivitelline membrane (PM), granulosa cells, and theca cell layers [33]. It contains only the inner layer of the PM since the outer layer of the PM is secreted at the start of the oviduct following ovulation. The internal PM exerts a similar role to the zona pellucida of mammalian follicles. There is more maternal mRNA within the avian oocyte than in mammals [34]. The ovulation involves the Hypothalamus-Pituitary-Gonadal (HPG) axis and the secretion of gonadotropin hormones (luteinizing (LH) and follicle-stimulating hormones (FSH)) [35]. Not all follicles develop at the same time [36,37,38]. When a follicle reaches the last stage of development, a trigger stimulus (the switching on or off the light, respectively in Japanese quail and hen) induces a LH surge, leading to the synthesis of progesterone by the mature follicle. Through positive feedback, progesterone (but not estradiol as observed in mammals) induces a second LH surge, called the pre-ovulatory surge. Significant secretion of ovarian steroids, including progesterone, accompanies the pre-ovulatory discharge. Progesterone allows for the release of enzymes responsible for the rupture of the follicular membrane, resulting in the release of the follicle into the oviduct.

**Figure 4:**
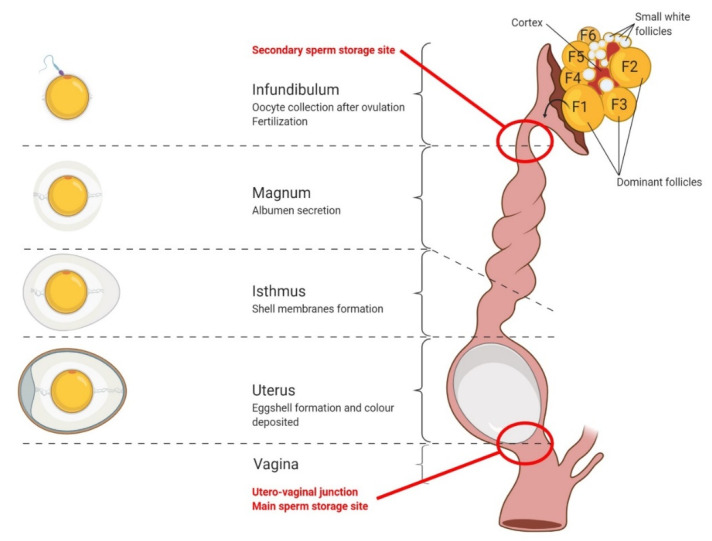
Structure of an avian oviduct with reference to sperm storage sites.

Unlike mammals, the late avian embryo development takes place outside the mother’s uterus. Thus, the egg must contain all the nutrients to allow for embryo development. The oviduct will allow for the formation of the egg but also its fertilization. The avian oviduct is made up of 6 successive anatomical area (Figure 4).

The infundibulum (also called pavilion), is located in the upper part of the oviduct. It captures the oocyte at the time of ovulation. Its wall is particularly thin and its mucosa contains several cell categories, some of which have a secretory function (deposition of proteins forming the perivitelline outer membrane of the egg), and for others, a storage function of sperm (infundibular glands). The infundibulum is the region where the egg is fertilized. The secretory activity of the infundibulum allows for the deposition of the outer layer of the perivitelline membrane. The completion of this thin membrane is made up of fibrils having a composition very close to that of gelatinous white egg. This composition plays an important role in the protection of yellow by limiting the exchanges between the latter and white egg.

The magnum, 30 to 35 cm long in an adult hen, is the area where the albumen (or white) is synthesized. In its lower part, the magnum is separated from the isthmus by a clear narrowing in diameter.

The isthmus is shorter (about 15 cm), and slightly narrower than the magnum. The internal folds of its mucosa are also less pronounced. Its color is very comparable to that of the magnum in the upper part and the mucosa becomes reddish in the area near the uterus. These two areas are called white isthmus and red isthmus, respectively.

The uterus (also called the shell gland), is the thickest part of the oviduct. More or less rounded in shape, its thick walls are surrounded by a highly developed musculature. The uterine lining, dark red in color, is formed-16-numerous folds covered with a surface epithelium, dotted with numerous branched tubular glands responsible for the secretion of the shell constituents. This lining is clearly distinguished from the utero-vaginal lining by the lack of orientation of the folds.

The utero-vaginal junction, only 1 to 2 cm long, is attached to the uterus by a thick fibrous structure. This junction plays an essential role in the prolonged storage of spermatozoa [39].

The vagina, about ten centimeters in length, is the most distal part of the oviduct and opens into the cloaca. It consists of a large layer of muscle tissue that allows for the final expulsion of the egg.

### 3.2. Fertilization

For fertilization, birds must copulate. Unlike mammals, most birds do not have external genitalia. Instead, they have cloacae, common openings for both reproduction and excretion. In most birds, copulation only lasts a few seconds, in which the male bird mounts the female from behind and their cloacae touch. The sperm swim up to the ovum where fertilization occurs.

Fertilization takes place in three stages [40]:

1. Penetration of the spermatozoon in the ovular cytoplasm;
2. Activation of the oocyte;
3. Fusion of the haploid nuclei of the two gametes and the reconstitution of a new diploid cell: the zygote.

In birds, after mating, the preservation of sperm takes place in the specialized tubular glands located at the base of the infundibulum and especially at the uterovaginal junction. Bird spermatozoa survive within the female genital tract and retain fertilizing power there for longer than that recorded in most mammals. This duration is however very different from one species of bird to another (from 4 to 30 days at least). The spermatozoa are stored within the oviduct in specialized tubular invaginations called sperm storage tubules [41] and released before subsequent ovulation by progesterone-mediated stimulation [42]. The penetration of the yolk (follicle) into the oviduct is favored by the infundibulum thanks to its funnel shape, animated by muscle contraction during ovulation and coming to cap the follicle ready to ovulate. The spermatozoa preferentially agglutinate in front of the germinal disc and insert between the fibers of the membrane. A tryptic enzyme named acrosin, released by the acrosome, allows for spermatozoa to penetrate. Fertilization in birds, including chickens, is different to mammals in that it requires multiple sperm to fertilize an egg cell. Indeed, polysperm is very common, but a single nucleus of sperm obviously fuses with the nucleus of the oocyte [43].

### 3.3. Early Embryo Development

**Figure 5:**
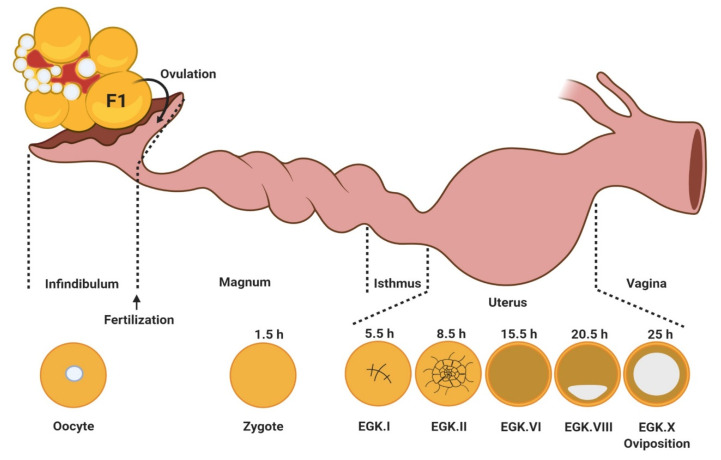
Some steps of early embryo development (intrauterine) in chicken.

After fertilization, the chicken eggs remain for about 3 h inside the magnum to acquire albumen, 1.5 h inside the isthmus to acquire shell membranes, and 20 h inside the shell gland to acquire a calcium-enriched shell. The zygote undergoes intrauterine embryo development with the initiation of cell division parallel to the egg position in the shell gland (Figure 5). The embryo development in birds, and particularly in chickens, is classified into stages by two major criteria: Eyal-Giladi and Kochav (EGK) criteria [44,45] and Hamburger and Hamilton (HH) criteria [46]. The EGK criteria classifies the intrauterine embryonic development, while the HH criteria classifies the post-ovipositional development in chickens. According to the EGK criteria, intrauterine embryo development in chickens is classified into stages from EGK.I (early cleavage stage) to EGK.X (showing complete formation of the area pellucida and area opaca) (Figure 5). After fertilization, shell formation, and egg laying, there is an incubation period, which varies in length for each species. During incubation, major embryo development occurs that we will not detail here. 

## 4. Adipokines and Their Receptors

Before describing the effects of adipokines on oocyte maturation, the fertilization, and the early embryo development processes, we will first briefly describe the gene and protein structure of the four main adipokines studied in this review (leptin, adiponectin, visfatin, and chemerin), their receptors in mammals, and then, their peculiarities in birds.

### 4.1. Leptin

**Figure 6:**
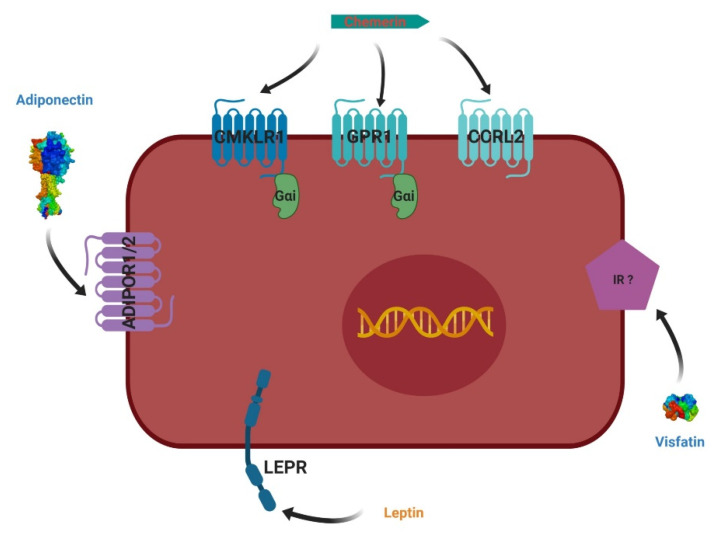
Leptin, adiponectin, visfatin, and chemerin and their receptors.

In mammals (humans), leptin is a peptide containing 167 aa (Table 1) that is known as a key regulator of satiety, metabolism, and energy homeostasis mainly in the central nervous system [47]. Six leptin receptor (LEPR) isoforms are generated by alternative splicing of the *LEPR* gene located on the chromosome (Chr) 7 (Table 1). LEPR-a, -b, -c, -d and -f are membrane-bound receptors but only LepR-b is full length with an intracellular signaling domain (Figure 6). Indeed, LEPR-b contains the intracellular domains necessary to mediate signal transduction through the signal transducer and activator of transcription 3 (STAT3) pathway [48,49]. Other pathways, including the mitogen-activated protein kinase [48,49], protein kinase C [50], and phosphoinositol 3-kinase pathways [51], are also activated by leptin. STAT3 signaling is required for leptin regulation of energy balance, but not for reproduction [52]. LEPR-b is the most important receptor in the hypothalamus and it is responsible for the control of energy intake and expenditure. LEPR-e is a soluble receptor, which binds circulating leptin.

In birds, the occurrence of the leptin gene had been a matter of debate over recent decades [63,64,65], but has recently been clarified by the finding of a true chicken leptin orthologue [66], which has been located on chromosome 1 [67] (Table 2). Chicken leptin and leptin receptor have an amino acid identity of 30% and 10% with human leptin and leptin receptor, respectively (Table 3). The hormone was reportedly expressed in non-adipose tissues [68,69] (Table 2). Indeed, as recently reported by Friedman-Einat and Seroussi, 2019, leptin is missing from the adipose tissue, observed under a variety of physiological and feeding conditions, indicating that in birds, leptin is not the signal by which the adipose tissue announces the amount of fat stores [70]. The dominant level of avian leptin receptor expression in the hypothalamic-pituitary-gonadal and -adrenal axes suggests its implication in the regulation of reproduction and stress response rather than appetite. However, further investigation is needed to establish this possibility. Chicken leptin was claimed to be undetectable in chicken blood [66]. Despite this, the availability of a functional chicken leptin receptor prompts studies targeting leptin receptor, which substantially augmented leptin bioactivities in both chicken hens and pullets [71,72]. 

### 4.2. Adiponectin

In mammals, the human adiponectin cDNA was isolated from WAT in 1996 by Maeda et al. (1996) as apM1 (adipose most abundant gene transcript 1 protein) [83] and in parallel, from murine fibroblast cell lines (ADIPOQ) by Hu et al. (1996) [84]. The adiponectin gene codes for a 26 kDa protein (244 aa) described for the first time by Scherer et al. (1995) [85] (Table 1). The adiponectin protein has also been extracted from human plasma [86], where it is considered to be the most abundant adipokine (between 5 and 30 mg/L). Adiponectin is secreted into the blood by white adipocytes with a higher serum level in women as compared to men and it is negatively associated with body weight. It is found in cells and plasma in three main forms: trimers, hexamers, and high molecular weight (HMW) [87]. In addition, a smaller fragment generated by the proteolytic cleavage of complete adiponectin generates a globular domain of the protein, gAd, which is secreted in plasma. Adiponectin is able to bind two main receptors, called ADIPOR1 and ADIPOR2, that have seven transmembrane domains, with a C-terminal extracellular region and an N-terminal cytoplasmic region [57] (Figure 6). These receptors, although ubiquitously expressed, have different tissue distributions. ADIPOR1 has a predominant localization in the skeletal muscles and the endothelial cells, while ADIPOR2 is mainly expressed in the liver. Adiponectin is also able to bind T-cadherin, which is a glycosyl-phosphatidylinositol receptor, belonging to the family of cadherins, which has no transmembrane domain. The intracellular signaling linked to this receptor seems to require other unidentified co-receptors or ADIPOR1 and ADIPOR2. In mammals, studies have shown beneficial effects of adiponectin on various physiological functions, including glucose homeostasis, food intake, apoptosis, and oxidative stress. This molecule is generally a beneficial adipokine [88,89]. 

In chickens, the adiponectin coding region shares 67% and 65% identity with humans and mice, respectively [75] (Table 3). Furthermore, the avian ADIPOR1 receptor cDNA sequence has 80–83% homology with the human, mouse, rat or pig cDNA sequences, while its deduced protein sequence is similar to 91% in mammalian forms. Similarly, the avian cDNA sequence of the ADIPOR2 receptor is 76–78% homologous with the human (Table 3), mouse or pig sequences, while its protein sequence is similar to 82% in the mammalian forms [77]. Adiponectin and its receptors are ubiquitous [76,90]. The expression of the adiponectin system (adiponectin, ADIPOR1, and ADIPOR2) in WAT and muscle depends on the sex and age of animals [91] (Table 2). In WAT, the expression of the adiponectin transcript is higher in females than in males, while the transcript of ADIPOR1 is higher in males than in females. In muscle, adiponectin and ADIPOR2 are more expressed in females than in males, and the opposite is observed for ADIPOR1 [82]. In addition, the adiponectin gene may be associated with the initiation and growth processes of WAT deposition in chickens [92,93].

### 4.3. Visfatin

In humans, the visfatin gene is found on the long arm of chromosome 7 and encodes for a secreted protein of 52 kDa [94] (Table 1). To date, no visfatin receptor has been identified (Figure 6). Visfatin is a pleiotropic protein involved in a broad spectrum of physiological processes, including metabolic functions [95]. In humans, visfatin was first discovered as a growth factor called PBEF (Pre-B cell colony enhancing factor) in 1994 from human peripheral blood lymphocytes capable of initiating the maturation of the precursors of B cells [94]. Visfatin is also considered to be a NAMPT (Nicotinamide phosphoribosyl transferase) type 2 due to its ability to synthesize NMN (mononucleotide nicotinamide) from nicotinamide and 5′-phosphoribosyl-1′-pyrophosphate. NMN is a therapeutic target for the treatment of metabolic disorders by improving glucose clearance in obese and diabetic mice [96,97,98].

In chickens, the full length of the visfatin gene has been cloned from adult liver. Avian visfatin protein has strong amino acid sequence similarities to that of humans (94%) Table 3), rodents (94%) [80], and other agronomical species (94%) [99]. Avian visfatin mRNA has been detected in many tissues such as the brain, heart, intestine, kidneys, liver, lungs, muscles, and spleen [80,100] (Table 2). Visfatin is also expressed in WAT without any difference between subcutaneous WAT and visceral WAT in chicken [80]. There is increasing evidence in birds that visfatin is involved in the regulation of muscle growth [101], metabolism [102], and food intake [103,104]. Thus, unlike mammals, visfatin is more a myokine than an adipokine in chicken.

### 4.4. Chemerin

In mammals, chemerin is a cytokine which is also known as TIG2 (tazarotene-induced gene 2) or RARRES2 (retinoic acid receptor responder 2) [105] (Table 1). It is an adipokine closely linked to the pathogenesis of metabolic syndromes [106]. Chemerin is secreted in the form of an inactive prochemerin of 143 amino acids, which is then hydrolyzed by the enzymatic cleavage of 5 to 7 amino acids from its carboxyl end in the extracellular compartment. Two neutrophil serine proteases, elastase and cathepsin G, eliminate 6 and 7 amino acids, respectively, to generate an active form. Plasmin and tryptase are also able to cleave 5 amino acids from the carboxyl end after cleavage of the carboxy-terminal lysine by carboxypeptidases N and B, which also lead to active chemerin [61]. Chemerin is secreted by fat adipocytes and it is expressed by several tissues, mainly WAT, liver and pancreas, but also placenta, skin, kidneys, adrenal glands, lungs, and intestines [107,108,109,110,111] (Table 1). Chemerin exerts its physiological functions by binding three receptors coupled to G proteins: CMKLR1 (chemokine like receptor 1), GPR1 (G protein coupled receptor 1) and CCRL2 (CC motif chemokine receptor like 2) [107,108] (Figure 6). CMKLR1 is coupled to the Gi/o family of G proteins and inhibits the cAMP signaling pathway (cyclic adenosine monophosphate), while promoting the PLC (phospholipase C), PI3K, and MAPK pathways, calcium mobilization [61], and the recruitment of β-arrestin which activates MAPK ERK1/2 (Extracellular signal-Regulated Kinases 1 and 2) [112]. The sequence of GPR1 is closely linked to that of CMKLR1 with more than 40% identity and activates the same signaling pathways [113]. However, CCRL2 does not seem to promote any signaling pathway, and does not induce the internalization of receptors [112]. Chemerin is involved in the regulation of blood pressure, inflammation, immune responses, differentiation of adipocytes, and carbohydrate metabolism, and plays a key role in metabolic diseases such as obesity and diabetes [10].

In birds, very few data are available. In turkeys, chemerin mRNA is mainly found in the liver compared to the heart and muscles, while CMKLR1 and GPR1 mRNAs are ubiquitous. The messenger of CCRL2 is strongly present in the pectoralis muscle compared to the liver, heart, and leg muscle (Table 2). The chicken chemerin gene sequence shares 81% identity with the turkey chemerin sequence. Chicken chemerin, CMKLR1, and GPR1 have an amino acid identity of 36%, 56%, and 64% with human chemerin and its receptor, respectively (Table 3). These results suggest that the chemerin system could act on the avian carbohydrate and lipid metabolism.

## 5. Involvement of Adipokines in the Oocyte Maturation, Fertilization, and Early Embryo Development in Mammals and Birds

In mice, genetic transformations of some adipokines or adipokines receptors genes induce female infertility or subfertility (Table 4). This could point to an essential role of these hormones in the hypothalamo-pituitary-ovarian axis regulation. For example, female mice with leptin KO are sterile [114]. This sterility can be easily corrected by injections of human recombinant leptin [115] or ovarian transplantation [116]. However, a lot of studies brought evidence that leptin, but also other adipokines, could exert specific roles in oocyte maturation, fertilization, and early embryo development in various species.

### 5.1. Leptin

#### 5.1.1. Oocyte Maturation

Leptin protein has been detected in the human, mouse, goat, and bovine oocytes ([121,122,123] (Table 5), whereas some authors failed to show its mRNA [124,125], suggesting it may be produced elsewhere and transported into the oocyte. *Lep-R* mRNA [124,126] and protein are found in mouse, rat, goat, and bovine germinal vesicle (GV) and metaphase II (MII) oocytes, suggesting that the oocyte is sensitive to leptin produced locally within the follicle (Table 5).

**Figure 7:**
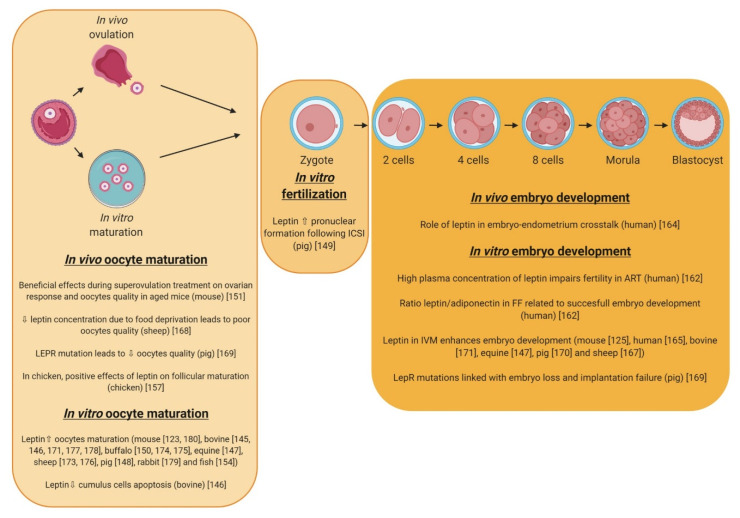
Effects of leptin on in vivo or in vitro oocyte maturation (IVM), in vitro fertilization, and in vivo or in vitro embryo development in different species. ICSI: Intracytoplasmic Spermatozoon Injection FF: Follicular Fluid. ART: Assisted Reproductive Technology.

In mice, leptin administration in in vitro culture medium increases the rate of meiotic resumption in preovulatory follicle-enclosed oocytes (Figure 7) [123]. Later study showed that leptin acts directly on the bovine oocyte via cumulus cells to enhance meiotic maturation [142,143]. These beneficial effects are associated with lower cumulus cells apoptosis due to leptin treatment and differential regulation of apoptosis-associated genes and genes involved in leptin signal transduction [144]. In vitro studies also showed that the addition of leptin to IVM medium enhances meiotic maturation and embryo development from calf oocytes and improves the quality of embryos derived from these oocytes [143]. Similar results showed that leptin treatment improves oocyte maturation and fertilization rates after ICSI in equine species [145]. It is well known that oocyte developmental potential is a reflection of proper nuclear and cytoplasmic maturation. In pigs, leptin enhances in vitro oocyte nuclear and cytoplasmic maturation via the Mitogen-Activated Protein Kinase Pathway [146]. It also enhances spindle assembly and accelerates pronuclear formation following fertilization [147]. This positive effect on oocyte nuclear maturation has also been described in buffalo species [148]. Joo et al. 2010 showed that leptin administration with gonadotropins during superovulation in aged mice increases the ovarian response, developmental competence of oocytes, and ovarian VEGF expression, suggesting that the promoting effect of leptin on oocyte quality may be mediated by increased VEGF expression within the ovary [149]. VEGF is well known to play a critical role in angiogenesis, and dysfunctional ovarian angiogenesis is supposed as one of the major causes of age-related decline of oocyte quality. Thus, the stimulation of angiogenesis by leptin-induced VEGF could contribute to the improvement of oocyte quality in aged mice [150,151]. In fish, a recent study shows that fatty acid β-oxidation is essential for leptin-mediated oocyte maturation [152]. Even if the role of lipid metabolism in oocyte maturation is increasingly recognized in mammals [153], the link between fatty acid β-oxidation and leptin effects remains to be demonstrated in mammals and birds. In chickens, leptin attenuates the negative effects of fasting on ovarian function. Injections of leptin during fasting delay cessation of egg laying and attenuates regression of yellow hierarchical follicles [154]. Cassy et al. suggest that leptin controls positively follicular maturation [155].

Thus, in many mammalian species, leptin has positive in vitro effects on oocyte maturation by reducing apoptosis and minimizing cellular damage to oocyte and/or cumulus cells, increasing ovarian angiogenesis and fatty acid oxidation. After fertilization, leptin improves pronuclear formation that is crucial for a good embryo development. As in mammals, leptin could exert positive effect on follicular maturation in chicken.

#### 5.1.2. Fertilization and Embryo Development

In embryos of mouse, human, bovine, chicken, and rabbit species, leptin is present and has specific spatial localization [121,122,128,156,157,158] (Table 5). In these studies, authors demonstrated that after fertilization, leptin becomes differentially distributed among the inner and outer blastomeres of the morula stage embryo in a pattern that persists with respect to the ICM and trophoblast through the hatched blastocyst stage, suggesting an important role of leptin in early embryo development. In humans, the ratio between leptin and body mass index (BMI) appears to be highly predictive of in vitro fertilization (IVF) success [159]. Still, in humans, the ratio between leptin and adiponectin in follicular fluids of the preovulatory follicles, is related to successful in vitro embryo development [160]. Human endometrium produces leptin and leptin secretion is influenced by the blastocyst [161], suggesting that the actions of leptin may be important during the preimplantation and implantation time windows. Thus, the leptin system may play an important role in the crosstalk between the preimplantation embryo and the receptive endometrium during the human implantation process (Figure 7) [162]. Later, in vitro studies revealed that the addition of recombinant leptin to the embryo culture media promotes the development from 2-cell stage embryos to the hatched blastocysts in mice and humans [125,163]. These results are abolished by using an antibody against the extracellular domain of LEPR. Moreover, results showed that leptin significantly increased the total cell number of blastocysts with a highest effect in the trophectoderm suggesting a paracrine effect of this hormone. However, other studies suggested a contrary role of leptin on embryo development [164]. Indeed, authors showed that significantly fewer leptin-exposed than control embryos hatched by day 5 and by day 6 of development. In addition, cells of leptin-exposed day 5 blastocysts showed a higher rate of DNA fragmentation, which is a sign of apoptosis. These positive and negative effects seem to be dose- and stage-dependent. In sheep, the concentration-dependent regulation pattern was confirmed, although sheep embryos appeared to be more sensitive in responding to leptin [165]. In this same species, food deprivation linked with low leptin production induces lower oocyte quality [166]. In pigs, polymorphism in LEPR gene sequence induced leptin resistance in the Iberian pig breed itself leading to a lower ovulation rate, early embryo loss, and implantation failure of embryos [167]. Another study in pigs demonstrated that embryo development was stimulated when cultured in the presence of leptin, and development was further enhanced when leptin was present during both oocyte maturation and embryo development [168]. In bovine, leptin treatment during oocyte maturation improved developmental potential, resulting in increased development to the blastocyst stage with reduced numbers of apoptotic cells [169]. Further, increased LEPR mRNA levels were detected in blastocysts originating from oocytes treated with leptin. Authors concluded that physiological doses of leptin during oocyte maturation may have long-term effects on the expression of developmentally important genes in early embryos. In chickens, the mRNA expression of leptin receptor was identified in embryonic ovaries [131] as well as in developed ovaries [154,170]. These findings suggest that leptin may contribute in ovarian development during embryogenesis.

Thus, most of the in vitro studies demonstrated that leptin exerts beneficial effects on oocyte maturation and early embryo development. However, some data are sometimes contradictory. These controversies can be explained by different experimental conditions. A summary of the leptin effects described in the literature is shown in Figure 7 and Table 6 for more details concerning the doses used, cell type studied, timing and period of treatment of the dose, and the cell type used. However, these data remain to demonstrate under in vivo conditions.

### 5.2. Adiponectin

**Figure 8:**
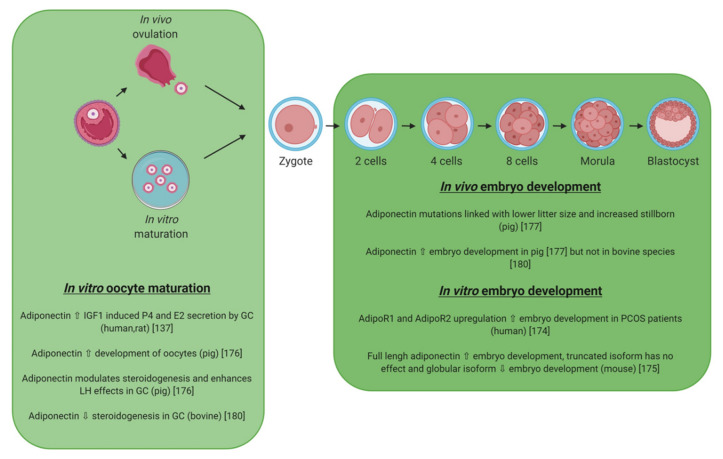
Effects of adiponectin on in vitro oocyte maturation, fertilization, and embryo development in different species. GC: Granulosa cells; P4: Progesterone; E2: Estradiol; PCOS: Polycystic Ovary Syndrome.

Adiponectin KO mice are subfertile (Table 4). In mice and rabbit species, adiponectin and its receptors ADIPOR1 and ADIPOR2 are expressed in blastocysts [133]. Its expression remains low or undetectable in granulosa cells in mice and human (Figure 8) [135,179]. In human, adiponectin increases IGF-1-induced P4 and E2 secretion in primary human GCs [135]. Adiponectin differentially regulates the expression of specific genes in granulosa cells and cumulus cells indicating that the effects of this adipokine are cell context specific and dependent on the stage of granulosa/cumulus cell differentiation [179]. As an example, adiponectin alone increases AdipoR1 and AdipoR2 expression in COCs but not in granulosa cells whereas it blocks the ability of FSH to increase AdipoR2 expression in both granulosa cells and cumulus cells. Adiponectin alone markedly suppresses the expression of Cyp11a1 in COCs but not in granulosa cells. It does not alter forskolin (FSH)- mediated induction of Cyp11a1 or granulosa cell release progesterone. In PCOS patients, the successful development of human early embryo is associated with the up-regulation of AdipoR1 and AdipoR2 [180]. These results suggest that adiponectin could positively modulates embryo development in humans. They are in a good agreement with the data of Richards et al., 2012, showing that adiponectin enhances in vitro oocyte maturation and in vitro early embryo development in mice and humans [179]. In mice, adiponectin can directly influence the in vitro development of the preimplantation embryo, and the effects are isoform dependent [181]. Indeed, the full-length adiponectin improves early embryo development, whereas the globular form has no effect, and the truncated form inhibits early embryo development. The adiponectin system (adiponectin, AdipoR1 and AdipoR2) is strongly expressed in rat theca cells, corpus luteum, and oocyte [132]. AdipoR2 and mainly AdipoR1 are also present in rat granulosa cells and enhance IGF-I-induced steroidogenesis. In porcine species, adiponectin, AdipoR1 and AdipoR2 are expressed in the cumulus cells, the oocytes from large follicles, and also in the early embryos [182]. Moreover, recombinant human adiponectin has a positive effect on oocyte meiotic maturation through the activation of the p38MAPK pathway and on the in vitro embryo development in pigs [182]. In granulosa cells, adiponectin modulates steroidogenesis by inhibiting Star expression and by stimulating p450scc expression. Moreover, it enhances LH effect on periovulatory genes expression by stimulating COX2 and PGES expressions through the MAPK ERK1/2 signaling pathway. In this species, several SNPs for adiponectin and its receptors have been identified as associated with a lower litter size and increased stillborn [183]. It appears also that adiponectin could be involved in a transgenerational effect of maternal undernutrition with a downregulation of AdipoR1 and an upregulation of AdipoR2 in early embryo as DNA methylation levels in embryos may be altered in response to female nutritional restriction [184]. Results are quite different in bovine species since this adipokine decreases insulin-induced steroidogenesis and increases IGF-1-induced proliferation of cultured GC through a potential involvement of the ERK1/2 MAPK pathway. However, it did not modify in vitro oocyte maturation and embryo development [185]. Finally, in birds, adiponectin, AdipoR1 and AdipoR2 mRNAs are expressed in chicken theca and granulosa cells from preovulatory follicles. However, adiponectin is higher expressed in theca cells than granulosa cells and could be involved in the development of chicken preovulatory follicle and oocyte maturation [186].

### 5.3. Visfatin

**Figure 9:**
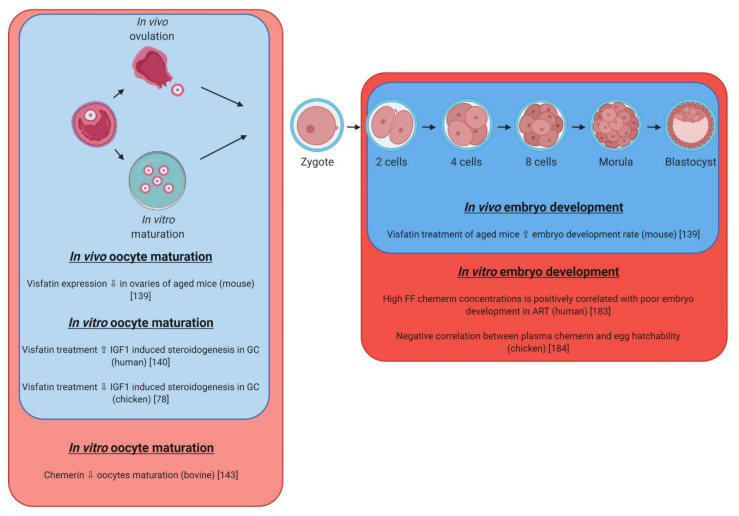
Effects of visfatin (in blue) and chemerin (in red) on in vivo and in vitro oocyte maturation, fertilization, and embryo development in different species. GC: Granulosa cells; IGF1: Insulin Growth Factor 1; FF: Follicular Fluid; ART: Assisted Reproductive Technology.

Very little information is available about the role of visfatin in early embryo development. The KO mice for visfatin are not viable since the embryos die at day 10 after conception [187], making harder the study of this adipokine. In vivo study demonstrated that visfatin expression declines in the ovaries of old mice but visfatin administration significantly increases embryo developmental rate (Figure 9) [137]. Visfatin-administered aged mice deliver significantly higher numbers of offspring than controls. Authors suggest the possibility that the promoting effect of visfatin on oocyte quality and fertility may be associated with the stimulation of ovarian angiogenesis. This adipokine has also an indirect beneficial effect on the embryo quality since it has been demonstrated that visfatin increases IGF-1-induced steroidogenesis and cell proliferation [138]. In birds, visfatin is expressed in the ovarian follicles of hens and it has a negative impact on steroidogenesis and P4 production by granulosa cells, and so, it could play a negative role on oocyte maturation [78]. However, until now, there is no evidence that visfatin has a direct action on early embryo development in mammals and birds.

### 5.4. Chemerin

A role for chemerin in early embryo development remains to be demonstrated. In human species, a recent study highlighted a relation between chemerin concentration in follicular fluid and embryo quality after IVF procedures. It appears that in PCOS women, the chemerin system is significantly overexpressed compared to the control and this is associated with the poorest embryo quality [188]. This adipokine has also a negative impact on in vitro oocyte maturation in bovine species (Figure 9) [141]. In chicken, it appears that chemerin concentrations in plasma are negatively correlated with egg hatchability, suggesting a potential role of this adipokine on egg [189]. In turkey, chemerin is expressed by granulosa cells and increases in the mature follicle before ovulation, thus, potentially influencing the EED [79].

## 6. Conclusions

**Figure 10:**
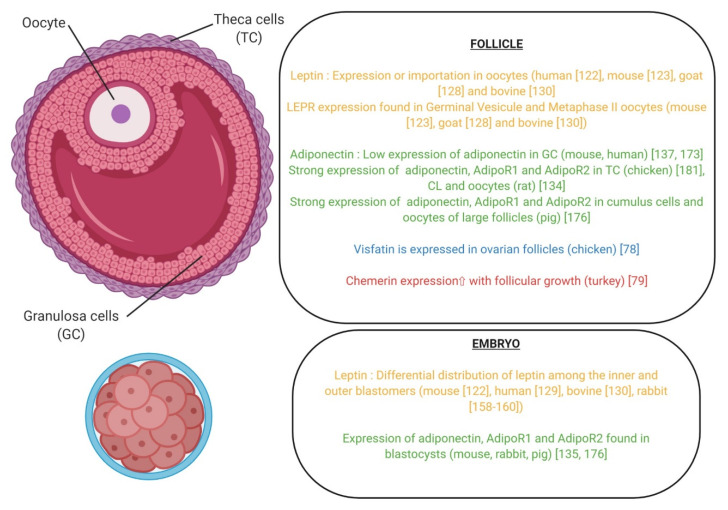
Expression of adipokines and their receptors in the ovarian follicle and embryo.

Adipokines (leptin, adiponectin, chemerin and visfatin) and their cognate receptors (LEPR for leptin, ADIPOR1, ADIPOR2 for adiponectin and CMKLR1, GPR1, CCRL2 for chemerin) are expressed in the oocyte and in the embryo of mammalian and avian species (Figure 10). In mammals, leptin exerts beneficial effects on IVM and embryo development in numerous species (Figure 7, Table 6). Similar data are shown for adiponectin in pigs (Figure 8), whereas chemerin reduces significantly IVM in bovine (Figure 9). Until now, even if total visfatin invalidation in mice leads to embryo lethality, no studies demonstrated a direct action of visfatin on IVM or/and early embryo development (Figure 9). Regarding data concerning the expression of adipokines in the blastocyst, the literature supports potential paracrine interactions/dialog between embryo and the mother via adipokines in early pregnancy. Indeed, several studies also show that adipokines and their receptors are present in the endometrial tissue. Thus, variation of adipokines or/and adipokine receptor expression or/and adipokine receptor signaling could be involved in embryo implantation. Concerning fertilization, not only the content in adipokines in egg but also those in spermatozoon have to be considered. Indeed, recent studies show potential effects of leptin and new adipokines on the quality of sperm [11,12]. In birds, and more precisely in chicken, a positive effect of leptin has been suggested on follicle differentiation whereas opposite data have been found concerning visfatin. No data are available concerning the role of chemerin and adiponectin on IVM and embryo development. The avian egg presents an opportunity to directly manipulate the developmental environment and study the role of the adipokine on embryo development via in ovo injections. The main problem is the lack of specific avian tools (antibodies, recombinant molecules, etc.,) to investigate the effects of adipokines in this species. Indeed, as showed in this review, adipokines in humans or rodents and chicken have low amino acid sequences identities. Finally, all the data suggest that additional studies are necessary to better understand the role and the molecular mechanism of adipokines in IVM and embryo development in mammals and birds, in order to determine their potential involvement in fertilization failures and embryo development disorders.

## Figures and Tables

**Table 1:** Gene location, protein expression, and synthesis of adipokines and its receptors in mammals (humans).

Protein (Name)	Gene	Gene Location	Protein (Description)	Synthesis	References
Leptin	*LEP/OB*	7q32.1	167 aa	WAT but also placenta	[53]
Leptin Receptor	*LEPR*	1p31.3.	1.165 aa (6 isoforms, a to f). LepR-b has intracellular signaling. LepR-e is a soluble receptor and binds plasma leptin.	LepR-b: strongly expressed in hypothalamus, but also in skeletal muscle. Ubiquitous	[54,55]
Adiponectin	*ADIPOQ*	3q27.3.	244 aa Full-length and Globular adiponectin (f and gADN) Three types of complex:Low molecular weight (LMW, 67 kDa), Middle molecular weight (MMW, 136 kDa), High molecular weight (HMW, >300 kDa).	Adipocytes	[56]
ADIPOR1	*ADIPO R1*	1q32.1	375 aa	Skeletal muscles and ubiquitously	[56,57]
ADIPOR2	*ADIPO R2*	12p13.33	386 aa	Liver and ubiquitously	[56,57]
Visfatin	*NAMPT*	7q22.3	491 aa	ubiquitously	[58]
Chemerin	*RARRES2*	7q36.1	163 aa	White adipose tissue and liver	[59,60]
CMKLR1	*CMKLR1*	12q23.3	373 aa	ubiquitously	[60,61,62]
GPR1	*GPR1*	2q33.3	355 aa	ubiquitously	[60,61,62]
CCRL2	*CCRL2*	3p21.31	344 aa	ubiquitously	[60,61,62]

**Table 2:** Gene location, protein expression, and synthesis of adipokines and its receptors in birds (chicken).

Protein (Name)	Gene	Gene Location	Protein (Description)	Synthesis	References
**Leptin**	LEP	Chr 1 (1p)	198 aa	mainly in brain and pituitary	[67,70,73]
**Leptin Receptor**	LEPR	Chr8	1146 aa	ubiquitously	[74]
**Adiponectin**	ADIPOQ	Chr9	244 aa	mainly fat tissue, heart, stomach and skin and ubiquitously	[75]
**ADIPOR1**	ADIPOR1	Chr32	376 aa	ubiquitously	[76,77]
**ADIPOR2**	ADIPOR2	Chr1	387 aa	ubiquitously	[76,77]
**Visfatin**	NAMPT	Chr1	493 aa	ubiquitously	[78,79,80]
**Chemerin**	RARRES2	Chr2	162 aa	mainly liver (turkey)	[79]
**CMKLR1**	CMKLR1	Chr15	360 aa	ubiquitously	[79]
**GPR1**	GPR1	Chr7	420 aa	ubiquitously	[79]
**CCRL2**	CCRL2	Nd	nd	Pectoralis muscle and ubiquitously	[79]

Chr: chromosome, aa: amino acids.

**Table 3:** Amino acid sequence identity between chicken and human species.

Protein	Leptin	Leptin Receptor	Adiponectin	ADIPOR1	ADIPOR2	Visfatin	Chemerin	CMKLR1	GPR1	CCRL2
Identity %	30	47	57	91	82	94	36	56	64	nd
Accession number or ref.	[81]	P48357 and Q9I8V6	[82]	[77]	[77]	[80]	Q99969 and A0A0K0PUH6	Q99788 and A0A1D5P7P2	F1NYB0 and P46091	

nd: undetermined.

**Table 4:** The consequences on the female fertility of targeted or total disruption or overexpression of components of some adipokine signaling pathways.

Adipokine Component Modified	Genetic Transformation	Ovarian Consequences	References
**Leptin**	Total Knockout (KO)	no mature follicles or corpora lutea were detected suppression of ovarian folliculogenesis and increase in ovarian granulosa cell apoptosis	[117]
**Leptin-R**	-Total KO (db mice) -Conditional deletion (cells expressing LH-β)	-Reduction of ovarian functions that are not due to Leptin-R expression in ovary -Reduction of litter size	[118,119]
**Adiponectin**	-Total KO	-Reduction of oocytes retrieval, disruption of estrous cycle, elevation of atretic follicles number, and decrease in late folliculogenesis	[120]

**Table 5:** Examples of adipokines and adipokine receptors expression in oocyte and embryos in several species.

Protein	Oocytes	Embryos
Leptin	Mice (protein, [123]), Goat (protein, [127]), Human ([121,122]; Bovine [128]	Mouse and Human [122], Bovine [128], Rabbit [129], Chicken [130]
Leptin receptor	Rodent (protein, [123]), Goat (protein [127]), Bovine [128]	Chicken [131], Bovine [128]
Adiponectin	Rat [132], Bovine [9]	Rabbit [133], Chicken [81]
Adiponectin Receptors	Rat [132], Bovine [9], Pig [134], and Human [135]	Rabbit [133], Chicken [81,136], Pig [134]
Visfatin	Rodent [137], Human [138], Bovine [139]	Chicken [140]
Chemerin	Bovine [141]	Chicken [140]
Chemerin receptors	Bovine [141]	Chicken [140]

**Table 6:** Description of experiments (cell type, species, time of incubation, dose and origin of leptin, etc.,) investigating the effect of leptin treatment on in vitro oocyte maturation and/or embryo development.

Cell Type	Species	Time of Incubation	Dose ng/mL	Origin of Leptin	Medium	Effects on Oocyte and/or Embryo	References
-Secondary follicle	sheep	18 days	25	human	α-MEM+	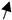 MII (%)	[171]
COCs	buffalo	24 h (IVM), 48 h (cleavage rate) and day 8 post IVF (blastocyst rate)	10	nd	TCM-199 (IVM) FerTALP (IVC)	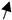 cleavage and blastocyst rate	[172]
COCs	buffalo	24 h (IVM)	10 and 50	mouse	TCM-199	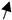 oocyte nuclear maturation	[173]
Preantral follicles and COCs	sheep	6 days and 24 h for IVM	10	human	TCM-199	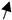 MII (%)	[174]
COCs	buffalo	24 h (IVM)	10	mouse	TCM-199	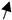 MII (%)	[148]
COCs	calf	24 h (IVM)	1 or 10	nd	TCM-199	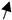 MII (%)	[143]
COCs	Prepubertal calf	24 h (IVM) 48 h (cleavage) 8 days (blastocyst)	10, 100 or 1000	human	TCM-199 (IVM)FerTALP (IVC)	No effect on cleavage and blastocyst levels	[175]
COCs	bovine	24 h (IVM) 7 days (blastocyst)	10, 100	human	TCM-199 FerTALP (IVC)	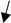 cleavage rate and blastocyst yield with leptin 100 ng/mL	[176]
COCs	rabbit	16 h (IVM)	1, 10, 100	nd	TCM-199	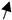 MII (%) (nuclear oocyte maturation)	[177]
COCs	horse	28 to 30 h (IVM)	100	human	TCM-199	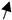 *in vitro* oocyte maturation	[145]
COCs	mouse	24 h (IVM)	10	mouse	M16	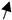 oocyte nuclear maturation	[178]
COCs	bovine	20–22 h (IVM)	1, 10	human	TCM-199	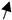 MII (%)	[144]
COCs	bovine	22–24 h (IVM) 7 days (blastocyst)	1, 10, 100	human	TCM-199FerTALP (IVC)	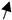 No effect on the cleavage rate but number of cells in blastocysts	[169]
COCs	pig	24–48 h (IVM)	10, 100, 1000	human	TCM-199	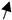 MII (%) with 10 and 100 ng/mL	[146]
Preovulatory follicle-enclosed oocytes	mouse	24 h (IVM)	10, 100, 1000	human	TCM-199	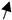 GVBD (%)	[123]

Notes: FerTALP: Tyrode-albumin-lactate-pyruvate fertilization media; IVC: in vitro embryo Culture, MII: metaphase II, GVBD: Germinal Vesicle Breakdown; IVM: in vitro maturation; COCs: Cumulus-Oocyte-Complexes; IVF: In Vitro Fertilization. 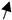: increase; 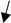: decrease.

## Abbreviations

ADIPOQ	Adiponectin
ADIPOR1	Adiponectin receptor 1
ADIPOR2	Adiponectin receptor 2
apM1	Adipose most abundant gene transcript 1 protein
BMI	Body Mass Index
CCRL2	CC motif chemokine receptor like 2
cDNA	Complementary deoxyribonucleic acid
CMKLR1	Chemokine like receptor 1
COCs	Cumulus-Oocyte-Complexe
COX2	Cytochrome c oxidase subunit II
EDD	Early embryo development
EGK	Eyal-Giladi and Kochav
E2	Estradiol
FSH	Follicule stimulating hormone
GPR1	G protein coupled receptor 1
GV	Germinal vesicule
hCG	human Chorionic Gonadotropin
HH	Hamburger and Hamilton
HMW	High molecular weight
HPG	Hypothalamus pituitary gonadal
ICM	Inner cell mass
IGF-1	Insulin like Growth Factor alpha
IVF	In Vitro Fertilization
IVM	In Vitro Maturation
KO	Knockout
LEPR	Leptin receptor
LH	Luteinizing Hormone
MAPK	Mitogen-Activated Protein Kinases
mRNA	Messenger Ribonucleic acid
NAMPT	Nicotinamide phosphoribosyltransferase
NMN	Mononucleotide nicotinamide
P4	Progesterone
P450ssc	P450 side-chain cleavage enzyme
PBEF	Pre-B-cell colony-enhancing factor
PCOS	Polycystic ovary syndrome
PGES	Prostaglandine E synthase
PI3K	Phosphoinositide 3-kinase
PM	Perivitellin membrane
RARRES 2	Retinoic Acid Receptor Responder protein 2
STAT3	Signal transducer and activator of transcription 3
SNPs	Single nucleotide polymorphisme
TE	Trophectoderm
TIG2	Tazarotene-induced gene 2
VEGF	Vascular endothelial growth factor
WAT	White adipose tissue
ZP	Zona pellucida
